# Mediastinal Follicular Dendritic Cell Sarcoma With Underlying Sjogren’s Syndrome

**DOI:** 10.7759/cureus.37715

**Published:** 2023-04-17

**Authors:** Davong D Phrathep, Kevin D Healey, Stefan Anthony, Kaila R Fives, Mitchell C Boshkos, Ruple Galani

**Affiliations:** 1 Medicine, Lake Erie College of Osteopathic Medicine, Bradenton, USA; 2 Internal Medicine, Lake Erie College of Osteopathic Medicine, Akron, USA; 3 Cardiology, Baptist Medical Center Beaches, Jacksonville, USA

**Keywords:** multicentric castleman disease, rare autoimmune disease, marginal zone b cell lymphoma, primary sjogren’s syndrome, follicular dendritic cell sarcoma

## Abstract

Follicular dendritic cells help advance B-Cells in becoming memory B-Cells or antibody-producing plasma cells in the light zone, or undergo additional affinity maturation in the dark zone. Follicular dendritic cell sarcoma (FDCS) is an extremely rare soft tissue malignancy derived from follicular dendritic cells. Autoimmune disease increases the risks for the development of hematological malignancies. To the best of our knowledge, there are few cases of FDCS development in the setting of underlying Sjogren’s syndrome (SS). Therefore, in this report, we present a novel case of FDCS associated with new-onset SS. In SS, the follicular dendritic cells are organized within germinal centers within the glands it infiltrates and is involved in B-Cell development. Because FDCS is derived from follicular dendritic cells, our report postulates that the unregulated follicular dendritic cell proliferation that may occur in SS could increase the risk for FDCS. Due to this possible connection observed in our patient, we highlight FDCS as a differential diagnosis when considering soft tissue cancers. We urge additional research to outline and explore the possible pathologic link between SS and FDCS.

## Introduction

Follicular dendritic cells are immune cells derived from mesenchyme that can be found predominantly in lymphoid tissue but can be found throughout the human body [1]. These cells function to bind antigens and link antigens to complement forming immune complexes that can be presented to germinal center B cells to initiate and maintain the secondary or adaptive immune response [2]. Follicular dendritic cell sarcoma is a rare and aggressive malignancy that affects intranodal and extranodal follicular dendritic cells [3]. Extranodal involvement occurs more frequently than intranodal involvement, which has been reported in roughly 79.4% of cases [1]. Malignancy involving follicular dendritic cells is extremely rare, with only 809 known cases reported in existing literature from 1986 to June 2021 [1]. 

It is also known that follicular dendritic cells may contribute to autoimmunity via the display of autoantigens [4]. Researchers have linked follicular dendritic cells with certain autoimmune diseases such as rheumatoid arthritis and systemic lupus erythematosus [4]. Sjogren’s syndrome (SS) is a systemic autoimmune disorder that presents with keratoconjunctivitis sicca and xerostomia due to inflammation involving both the salivary and lacrimal glands [5]. In this case report, we present a novel case of follicular dendritic cell sarcoma (FDCS) associated with new-onset SS.

## Case presentation

A 72-year-old Caucasian male presented to his primary care office with a dry mouth, dry eyes, and a swollen lump below his right jaw for two weeks. The patient stated that he has a dry mouth and noticed a progressive decline in saliva production. Physical examination revealed right salivary gland enlargement without masses and tenderness to palpation. The patient was advised to alleviate his symptoms with a warm compress and salivary gland massages. A CT of the salivary gland was ordered and showed no abnormal mass, fluid collection, or enhancement under the right submandibular region. The salivary gland revealed normal morphology and signal intensity of the parotid and submandibular glands. At this moment, the patient was diagnosed with SS. On imaging, there were enlarged superior mediastinal lymph nodes incidentally noted with the largest measuring 5 cm x 3.90 cm. The etiology was determined as unknown but further evaluation was warranted. The chest CT without contrast revealed three large abnormal mediastinal lymph nodes with the largest measuring 5.2 cm in greatest dimension. Neoplasm was considered until proven otherwise. Post the chest CT findings, a positron emission tomography (PET)/CT skull-thigh was ordered to analyze the patient’s abnormal mediastinal lymphadenopathy due to clinical suspicion of B-cell lymphoma. The PET/CT skull-thigh revealed hypermetabolic lymphadenopathy within the right paratracheal and prevascular spaces of the mediastinum. There was no hypermetabolic adenopathy beyond the mediastinum. Additionally, a fluoroscopic-guided bone marrow biopsy of the patient’s left iliac bone revealed normal iliac bone anatomy. 

Seven months after the incidental mediastinal lymphadenopathy finding, the patient was admitted for surgical resection, and a complete blood count was collected (Table 1). A complete metabolic panel was also conducted (Table 2).

**Table 1:** Complete blood count of the patient at the time of admission MCV: Mean corpuscular volume, MCH: Mean corpuscular hemoglobin, MCHC: Mean corpuscular hemoglobin concentration, RDW: Red blood cell distribution width, MPV: Mean platelet volume

Component	Patient’s value	Reference range
WBC count	8.50	4.50 - 11.00 K/mcL
RBC count	3.25	4.30 - 5.90 mil/mcL
Hemoglobin	9.3	13.50 - 17.50 g/dL
Hematocrit	28.1	41.00 - 53.00 %
MCV	86.5	80.00 - 100.00 fL
MCH	28.6	25.00 - 35.00 pg
MCHC	33.1	30.00 - 37.00 g/dL
RDW	14.1	11.50 - 15.50 %
Platelet count	116	150.00 - 450.00 K/mcL
MPV	9.4	7.00 - 11.00 fL
Neutrophils %	81%	40.00 - 75.00 %
Lymphocytes %	12%	15.00 - 45.00 %
Monocytes %	8	3.00 - 13.00 %
Eosinophils %	0	0.00 - 7.00 %
Basophils %	0	0.00 - 2.00 %
Neutrophils (absolute)	6.80	1.80 - 8.00 K/mcL
Lymphocytes (absolute)	1.00	1.00 - 5.00 K/mcL
Monocytes (absolute)	0.60	0.20 - 0.90 K/mcL
Eosinophils (absolute)	0.00	0.00 - 0.45 K/mcL
Basophils (absolute)	0.00	0.00 - 0.10 K/mcL

**Table 2:** Complete metabolic panel of the patient at the time of admission BUN: Blood urea nitrogen, eGFR: Estimated glomerular filtration rate

Component	Patient’s value	Reference range
Sodium	133	135 - 145 mEq/L
Potassium	4.1	3.50 - 5.10 mEq/L
Chloride	104	98 - 110 mEq/L
Carbon dioxide	21	22 - 32 mEq/L
Glucose	132	74 - 109 mg/dL
BUN	21	7 - 23 mg/dL
Creatinine	0.86	0.70 - 1.50 mg/dL
Anion gap	8	8 - 16 mEq/L
Osmolality (calculated)	271	280 - 300 mOsm/kg
Calcium	8.2	8.50 - 10.50 mg/dL
eGFR Caucasian male	86.7	> = 60.0 mL/min
eGFR African American male	105.1	> = 60.0 mL/min
Calculated serum creatinine clearance	93.88	97 to 137 mL/min

A right video-assisted thoracoscopic surgery (VATS) resection of the mediastinal mass was completed along with multiple biopsy attempts. At this time, marginal zone lymphoma was determined. After the VATS procedure, the patient was discharged home without complications and began a chemotherapy regimen of bendamustine and rituximab for six cycles for eight months. 

In the following year after completing chemotherapy, an outpatient postoperative follow-up revealed progressive enlargement of the right paratracheal lymphadenopathy. The PET/CT scan showed an increase in size and metabolic activity of the right paratracheal lymphadenopathy measuring up to 3.4 cm x 3.1 cm (previously 2.2 cm x 2.1 cm), with maximum standardized uptake values (SUV) of 22.2 (previously 10.3). Additionally, the imaging revealed an increase in the size of a 17 mm x 12 mm prevascular lymph node with a maximum SUV of 2.8. 

Due to the enlarging right paratracheal adenopathy, the patient underwent right robotic-assisted partial resection of the paratracheal mass. The necrotic abnormal tissue was excised and submitted for frozen analysis, which confirmed lymphoma involving the right paratracheal mass. Additional tissue was submitted for fresh pathology, permanent pathology, and microbiology. A month later, the right paratracheal mass biopsy and immunohistochemistry highlighting podoplanin D2-40 (Table 3) revealed FDCS.

**Table 3:** Immunohistochemistry CD: Cluster of differentiation, TTF-1: Thyroid transcription factor-1, BCL: B-cell lymphoma, P63: Tumour protein 63, CK: Cytokeratin, Sox: Sry-related HMg-Box, SMA: Smooth muscle actin, HMB: Human melanoma black, Mart: Melan-A protein and melanoma antigen recognized by T cells, MPO: Myeloperoxidase

Component	Analysis
Pankeratin	Negative
CD 56	Negative
Synaptophysin	Shows faint staining
Chromogranin	Negative
Ki-67	Highlights scattered cells
TTF-1	Highlights scattered cells
CD 45	Highlights scattered lymphocytes (mostly T-Cells)
CD 3	Highlights T cells
CD 20	Negative
Pax 5	Negative
CD 79a	Highlights scattered plasma cells and B cells
CD 138	Highlights scattered plasma cells
CD 10	Negative
BCL 6	Negative
Vimentin	Highlights neoplastic cells
S100	Negative
CD 68	Highlights histiocytes
NapsinA	Negative
P63	Negative
CK 5/6	Negative
CAM 5.2	Negative
Sox 10	Negative
SMA	Negative
Desmin	Negative
HMB45	Negative
Mart1	Negative
Myogenin	Negative
MPO	Negative
Podoplanin D2-40	Highlights scattered cells

One month following the diagnosis of FDCS, the patient underwent genetic testing for cancer-type relevant biomarkers, genomic signatures, and pathogenic alterations. The patient’s tumor was positive for programmed death-1 ligand 1 (PD-L1) expression, a FANCM pathogenic variant, and a Janus kinase 1 (JAK1) pathogenic variant. Genomic signatures were analyzed and revealed no additional abnormalities. 

After genetic testing, the patient was advised to follow up with oncology. During this visit, the PET scan results and the treatment plan was reviewed with the patient. The patient’s case was discussed with the tumor board, and local therapy surgery +/- radiation therapy was recommended. At this stage, the literature supported surgical resection of the sarcoma as the main treatment approach and suggested that the role of adjuvant therapy was questionable. The patient was advised to follow up with radiation oncology to discuss the role of radiation therapy. Physical examination revealed no palpable adenopathy, B-symptoms, or respiratory symptoms. During the consultation, it was discussed that FDCS may be on a continuum of lymphoid lesions that are at increased risk in patients with SS. Because mantle zone lymphoma commonly appears in the salivary glands, it was suggested that the mediastinal lymphoma may have been related to FDCS initially. Combined surgery and radiation therapy were scheduled for a month from the radiation oncology consultation. An additional CT chest with IV contrast was ordered for sarcoma staging. Imaging revealed a slight interval increase in the size of anterior and middle mediastinal lymphadenopathy without any new sites of metastatic disease in the chest. Following sarcoma staging, cardiology was consulted to perform a preoperative cardiovascular exam. Based on the American College of Cardiology and American Heart Association (ACC/AHA) guidelines the patient was determined to be at moderate risk for a perioperative cardiovascular event during the planned right thoracotomy. And so, the patient accepted that he would be at higher risk for postoperative atrial fibrillation. At this stage, the patient was scheduled for a right thoracotomy for FDCS analysis and underwent a successful resection.

## Discussion

Monocytes and related-immune cells are divided into two types: (1) phagocytes, and (2) dendritic cells. The dendritic cells include follicular dendritic cells, interdigitating dendritic cells, intestinal dendritic cells, Langerhans cells, and indeterminate cells [6]. Follicular dendritic cells are mesenchymal-derived dendritic cells located in B-follicles, where they help trigger and maintain the B-cell adaptive immune response [1]. More specifically, follicular dendritic cells help advance B-Cells into germinal selection in the light zone where B-Cells will either leave the germinal center as memory B cells or antibody-producing plasma cells or, return to the dark zone for further affinity maturation [7]. Follicular dendritic cell sarcoma is a rare type of soft tissue malignant neoplasm derived from these antigen-presenting cells. It has an unknown etiology with only 809 cases reported in the English literature dating back to 1986 [1]. With a median age of 49 years old, this uncommon tumor infiltrates the cervical, mediastinal, and/or axillary lymph nodes with no gender distinction [1]. Patients with FDCS may present with painless lymphadenopathy, cough, sore throat, difficulty swallowing, weight loss, and/or fatigue. 

Due to its rarity, FDCS is frequently misdiagnosed and is often not considered in a differential diagnosis [1]. Histology and immunohistochemical staining are required when there is a high level of clinical suspicion in symptomatic patients. Typically, immunohistological staining of cluster of differentiation (CD)21, CD23, and CD35 protein markers confirm FDCS diagnosis [1]. Podoplanin D2-40 is a highly effective marker of follicular dendritic cells and was positive for our patient, which supported our diagnosis. Clusterin, a protein representative of cleared cellular debris and apoptosis, is highly sensitive and specific for FDCS [8]. Other markers that support the diagnosis of FDCS include CXCL13, follicular dendritic cell-secreted protein (FDCSP), terminal deoxynucleotidyl transferase (TdT), CD68, fascin, and/or claudin-4 [8]. Histologically, FDCS is composed of uniform, spindle, and ovoid cells with eosinophilic cytoplasm, distinct nucleoli, nuclear pseudoinclusions, and a vesicular chromatin pattern [1]. Neoplastic cells are typically arranged in fascicular, syncytial sheets with storiform, whorled patterns, and low mitotic rates in most non-pleomorphic cases as seen in [9]. 

Although FDCS usually has an indolent course, local recurrence is common. Nodal involvement occurs in 15% of cases, with 25 cases involving the mediastinum [1]. Around 79.4% of cases predominantly occur in extranodal sites, with 42 reported cases involving the mediastinum [1]. Common extranodal sites are the liver, the spleen, and the gastrointestinal tract. Nuclear atypia/pleomorphism, intra-abdominal in location, larger than 6 cm in size, greater than 5 mitoses per 10 high-power fields, or coagulative necrosis are poor prognostic factors [10]. Follicular dendritic cell sarcoma shows a two-year survival rate of 82.8% for local disease and a two-year survival rate of 42.8% for metastatic disease to the lung, liver, or lymph nodes [10]. Optimal treatment recommendations for patients with FDCS have not been well-established, however, most localized cases are treated by surgical resection, with or without adjuvant chemotherapy and/or radiation [1]. In the literature, patients who receive a combination of surgery, chemotherapy, and radiation have been found to have longer disease-free survival [11]. 

Castleman disease (CD) is a benign tumor that develops in the lymph nodes and is typically associated with abnormalities of follicular dendritic cells, especially in the hyaline-vascular histological subtype and in the multicentric variant [2]. The connection between FDCS and CD provides our case the foundation to identify additional diseases that may have novel associations with FDCS. To our knowledge, there have been few, if at all, cases reporting FDCS development with underlying SS. In this report, we present a 72-year-old male with newly diagnosed FDCS within two years of being diagnosed with SS. Sjogren’s syndrome is the second most common autoimmune disease affecting mainly middle-aged women. It is characterized by overstimulation of the immune system and interferons. Important symptoms of SS are xerostomia and keratoconjunctivitis sicca caused by lymphocytic infiltration and destruction of the exocrine glands, particularly the lacrimal and salivary glands [12]. Patients with SS have an increased risk of developing malignancies, particularly non-Hodgkin B-cell lymphoma like marginal zone lymphoma as seen in our patient [5]. Additionally, oral cancer, breast cancer, and thymoma might also occur in patients with SS.

Although SS elevates the risk of developing non-Hodgkin’s lymphoma [8], the association between FDCS and SS has not been well-defined in the literature. Based on our patient’s clinical course, we postulate that SS could potentially have an association with FDCSa. In SS, the follicular dendritic cells are organized into networks within germinal centers in severe lesions within the glands and are critically involved in B-Cell development. As FDCS is derived from follicular dendritic cells, we postulate that the unregulated follicular dendritic cell proliferation that may occur in SS could increase the risk for FDCS. Due to this mechanism, FDCS should be considered as a differential diagnosis with worsening symptoms in a patient with underlying SS. Our unique case brings attention to a rare instance of FDCS in an older-aged male with underlying SS.

## Conclusions

In this report, we discussed the unique case of a 72-year-old male who was diagnosed with FDCS, an exceptionally rare malignant soft tissue neoplasm that may be linked to his diagnosis of SS two years prior. Patients with autoimmune disease, specifically SS, are placed at greater risk for hematological malignancies. Due to this possible association, further research to definitively outline the possible pathologic link between SS and FDCS is needed. Currently, there exist only 809 cases of diagnosed FDCS in English literature. Follicular dendritic cell sarcoma ordinarily follows an indolent course, however, local recurrence is frequently seen. The vast majority of FDCS malignancies involve extranodal sites such as the liver, spleen, and gastrointestinal tract. The FDCS literature lacks definitive and optimal treatment regimens, however, surgical resection with adjuvant chemotherapy and radiation likely provide the best outcomes. In light of this unique case presentation, we hope to bring more light to FDCS as a differential diagnosis when considering soft tissue malignancies and hope to stimulate further research that explores the possible pathological link between SS and FDCS.
